# Supplementary material for: Ultrathin-metal-film-based transparent electrodes with relative transmittance surpassing 100%
Source: Nat Commun. 2020 Jul 6;11:3367. doi: 10.1038/s41467-020-17107-6 (PMC7338390; doi:10.1038/s41467-020-17107-6)
Supplement: Supplementary file 1 — Supplementary Information [file 41467_2020_17107_MOESM1_ESM.pdf]

Supplementary Information

Ultrathin-metal-film-based transparent electrodes with relative transmittance surpassing 100%

Ji *et al*.

Supplementary Figure 1

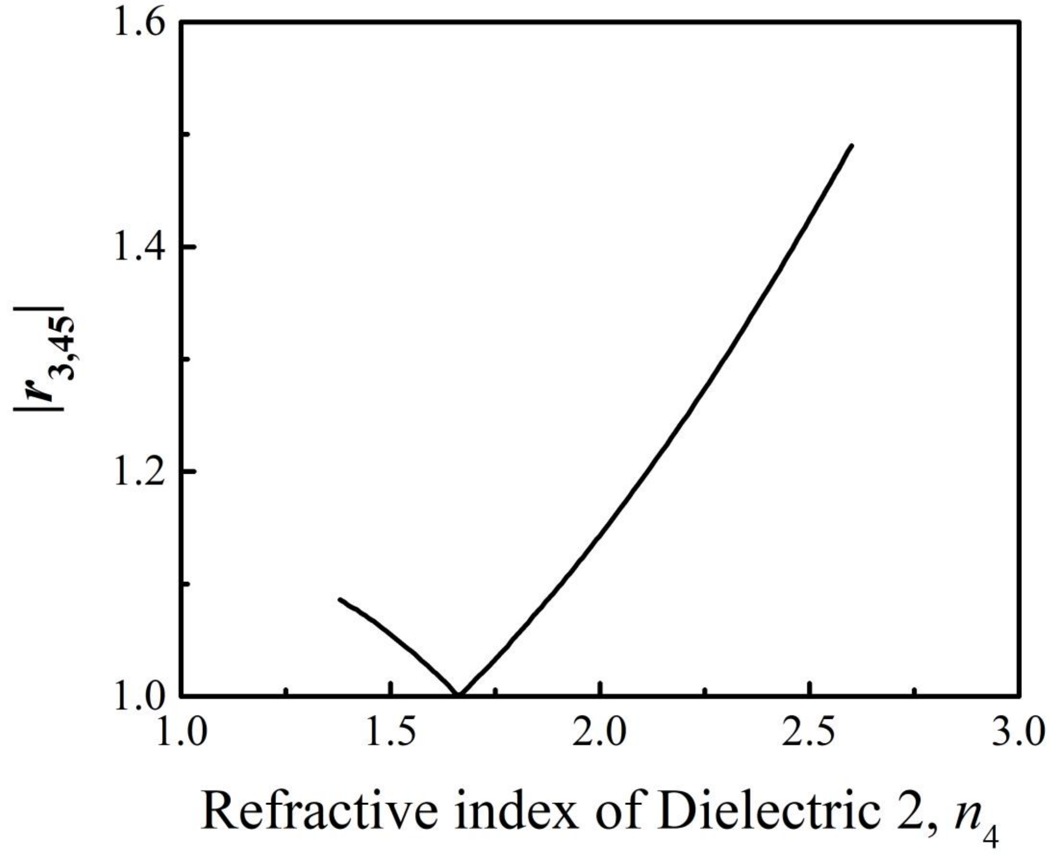

Supplementary Figure 1. Variation of $|r_{3,45}|$ with n_4, the refractive index of Dielectric 2. n_4 ranges from 1.38 (MgF_2) to 2.60 (TiO_2). The complex refractive indices of Cu-doped Ag and PET at 550 nm were used. The thickness of Dielectric 2 was determined using Equation (9). When n_4 is larger than the refractive index of PET, $|r_{3,45}|$ increases with n_4. $|r_{3,45}|$ is larger than one because medium 3 is metallic Cu-doped Ag.

Supplementary Figure 2

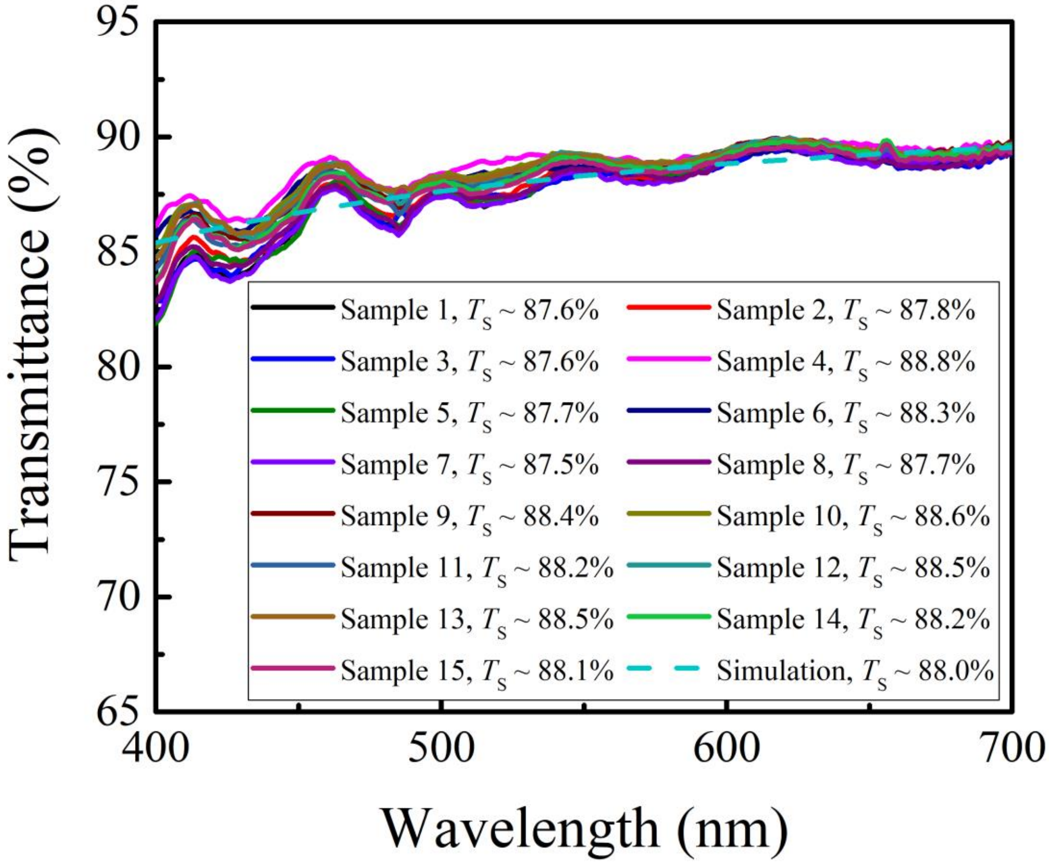

Supplementary Figure 2. Measured and simulated visible transmittance spectra of the PET substrate. The experimental averaged transmittance over the visible range (400 to 700 nm) is of ~ (88.1 ± 0.4) %, which is calculated with the measurement results of 15 samples. The cited uncertainty represents one standard deviation of the measured data. The theoretical averaged transmittance, which is calculated with the fitted refractive index of the PET substrate (refer to Supplementary Figure 6), shows an average value of ~ 88.0%, which is very close to the measurement result. The slight discrepancy is due to the small index fitting inaccuracy within the short wavelength range from 400 to 480 nm. Source data are provided as a Source Data file.

Supplementary Figure 3

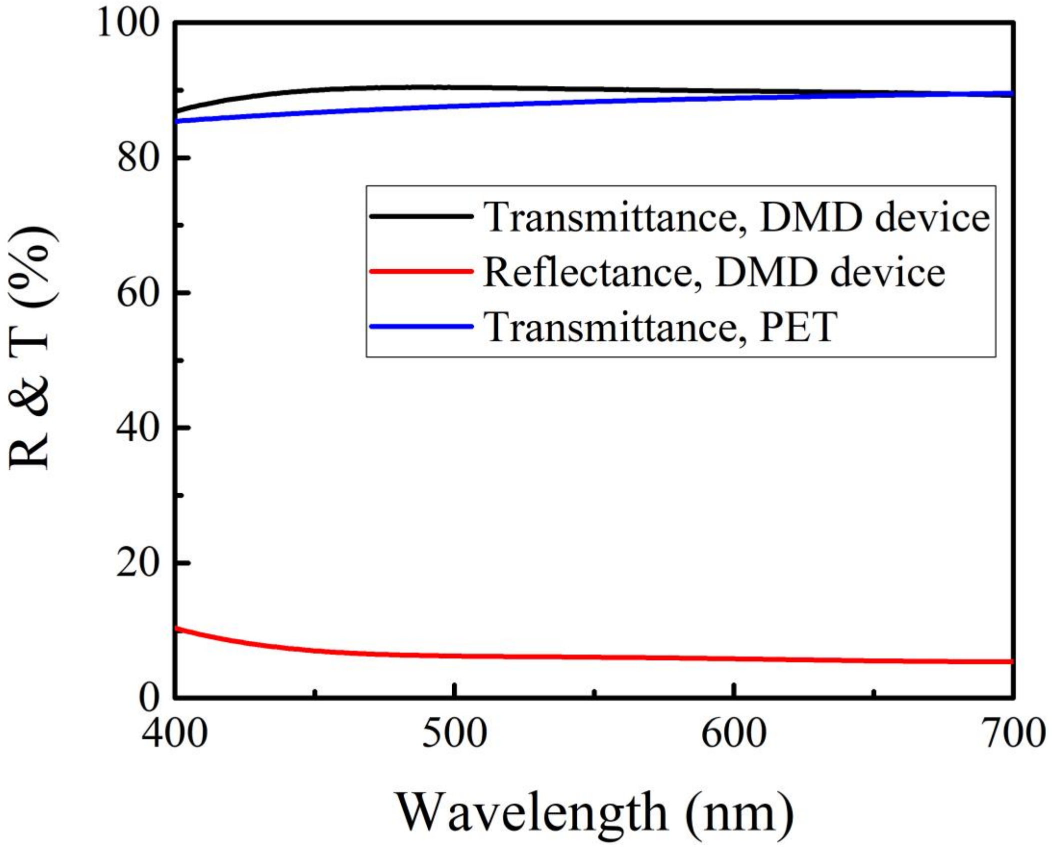

Supplementary Figure 3. Calculated absolute transmittance and reflectance spectra of the optimized structure 'PET substrate / 24 nm TiO_2 / 9 nm Ag / 63 nm Dielectric 1' across the entire visible range. Here, the refractive indices of TiO_2 and Dielectric 1 are assumed as 2.6 and 1.7, respectively. The averaged transmittance and reflectance are of ~ 89.8% and ~ 6.4%, respectively. The simulated transmittance spectrum of the PET substrate is provided as a reference, showing an averaged transmittance from 400 to 700 nm of ~ 88.0%. It is clear that the transmittance of the DMD electrode is well above that of the substrate especially at wavelengths < 600 nm.

Supplementary Figure 4

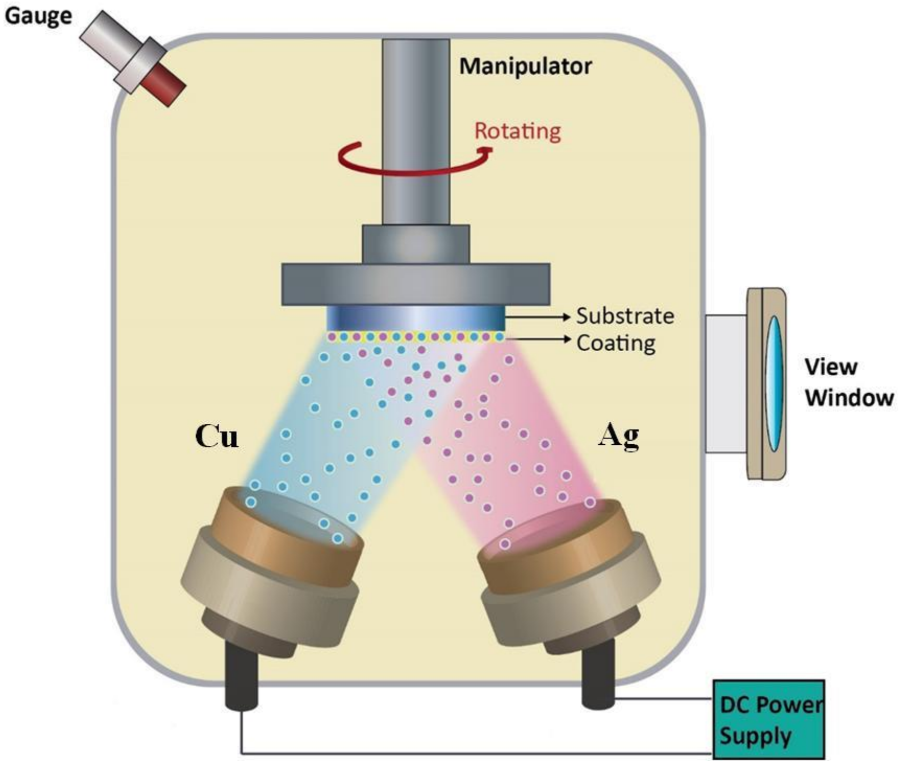

Supplementary Figure 4. Schematic of the co-sputtering system from Cu and Ag targets. More details of the deposition system can be found in our earlier work[1].

Supplementary Figure 5

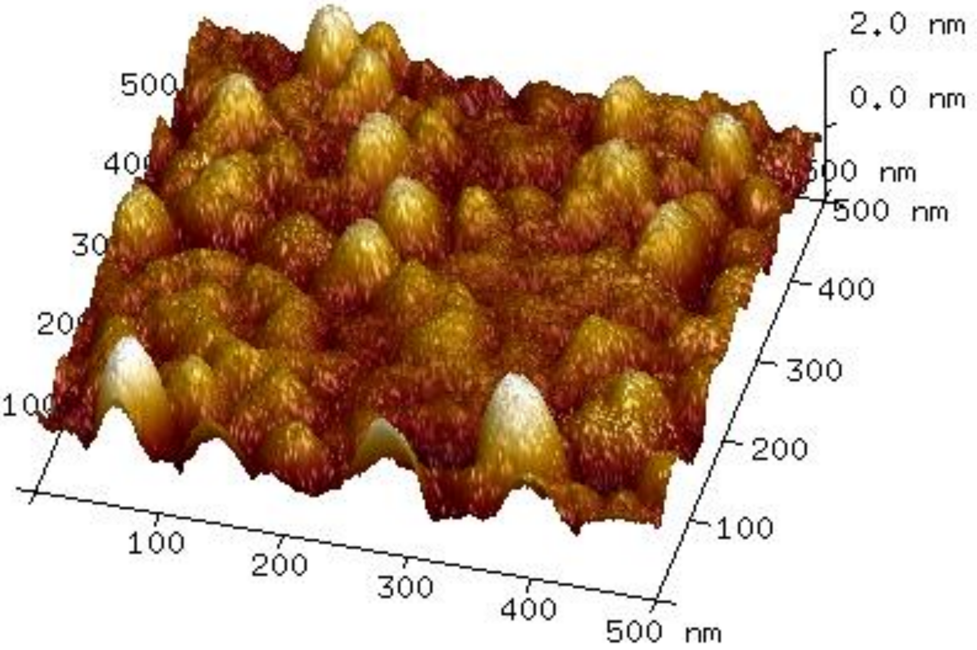

Supplementary Figure 5. 3D view of the atomic force microscopy (AFM) scan of the 6.5-nm-thick Cu-doped Ag film deposited on a fused silica substrate. The root-mean-square (RMS) roughness of the film is measured to be ~ 0.47 nm.

Supplementary Figure 6

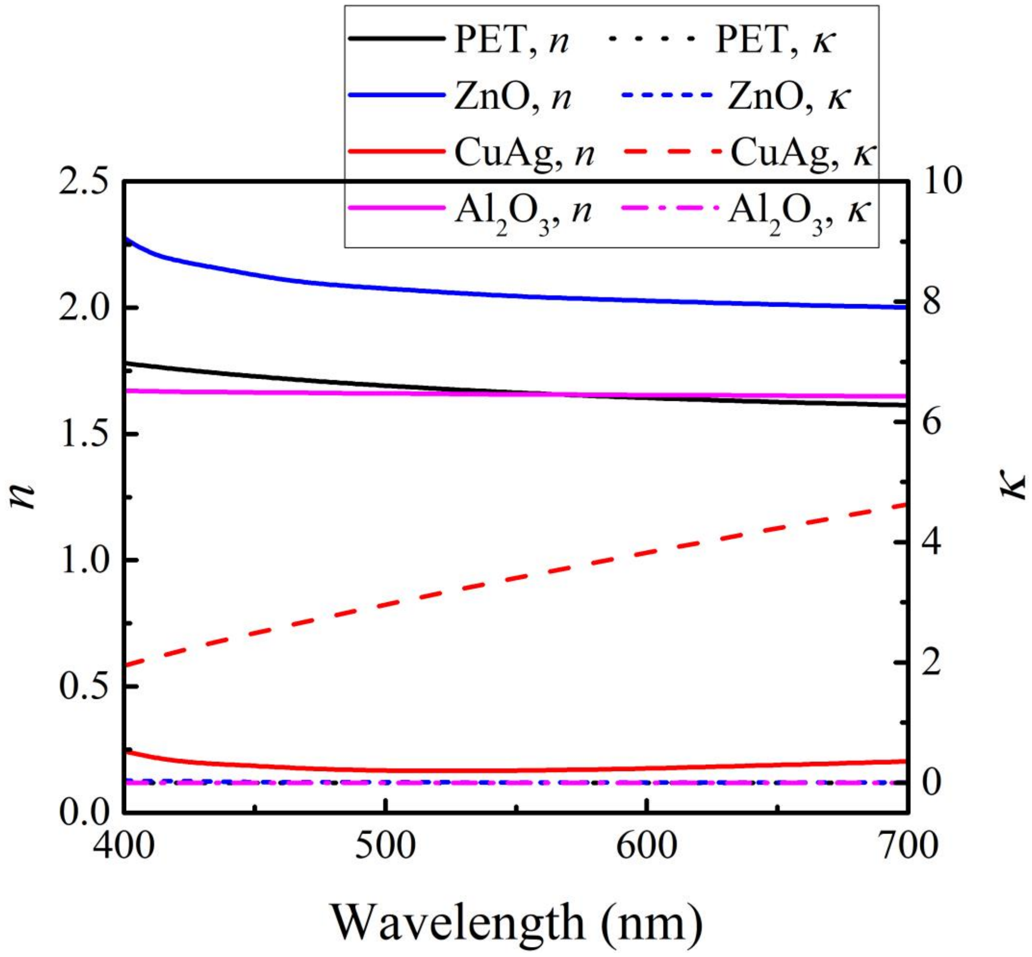

Supplementary Figure 6. Refractive index (*n*) and extinction coefficient (*κ*) of the PET substrate, ZnO, Cu-doped Ag, and Al_2O_3.

Supplementary Figure 7

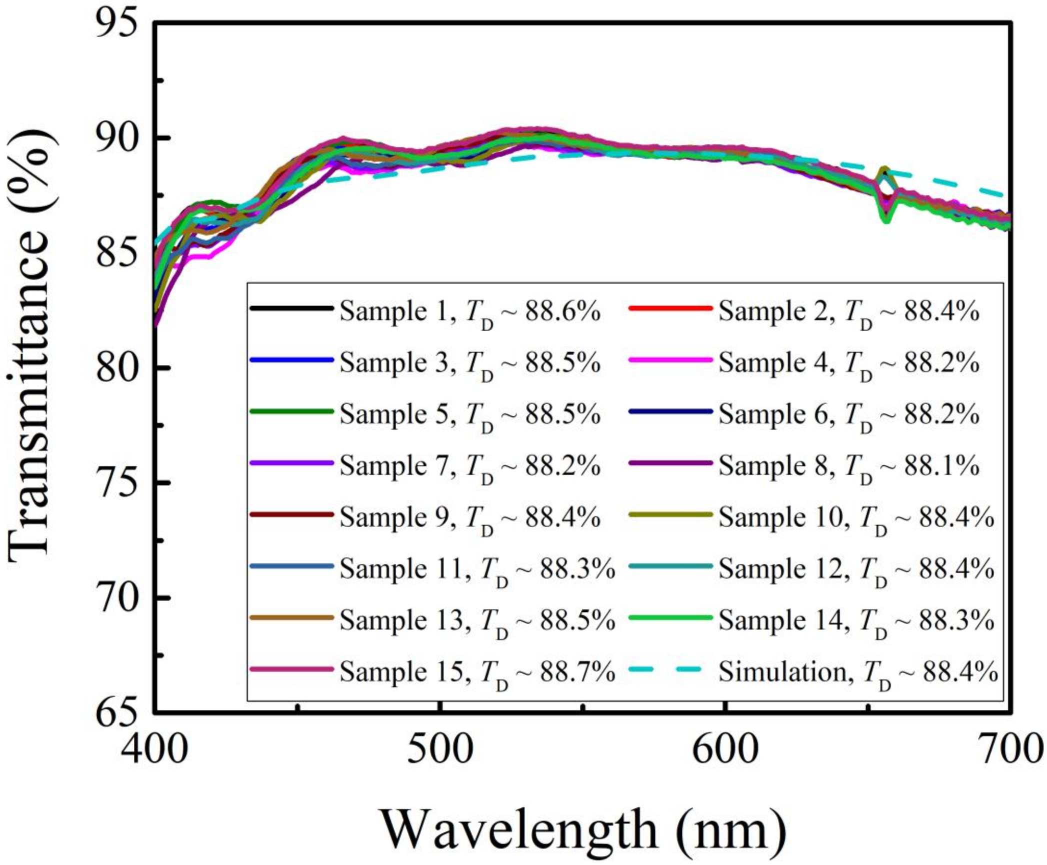

Supplementary Figure 7. Measured and simulated visible transmittance spectra of the designed DMD electrode on the PET substrate. The experimental averaged transmittance over the visible range (400 to 700 nm) is of ~ (88.4 ± 0.1) %, which is calculated with the measurement results of 15 samples. The cited uncertainty represents one standard deviation of the measured data. The theoretical averaged transmittance, which is calculated with the fitted refractive indices of all the materials (refer to Supplementary Figure 6), shows an average value of ~ 88.4%, which agrees well with the measured data. Source data are provided as a Source Data file.

Supplementary Figure 8

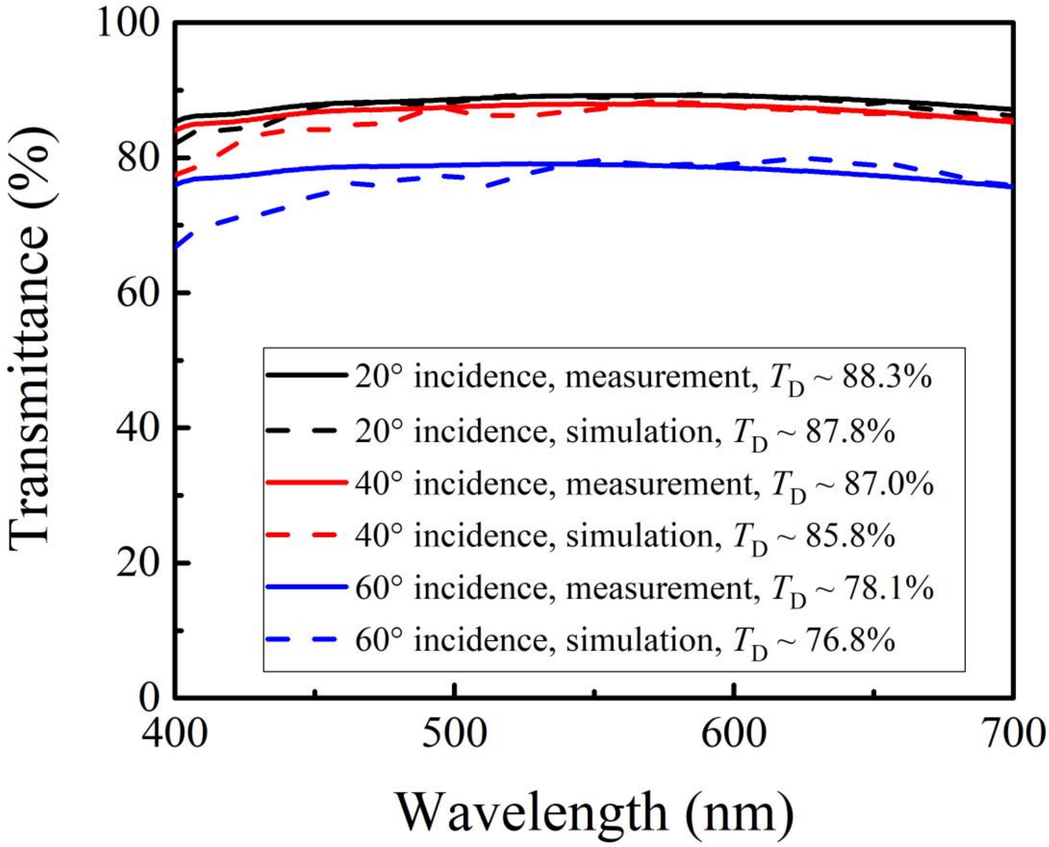

Supplementary Figure 8. Measured and calculated transmittance spectra of the DMD electrode on the PET substrate at different incident angles. High transmittance > 75% is maintained up to 60° angle of incidence. The slight discrepancy between the simulations and measurements at short wavelengths is due to the small index fitting inaccuracy of the PET substrate, which is discussed in Supplementary Figure 2.

Supplementary Figure 9

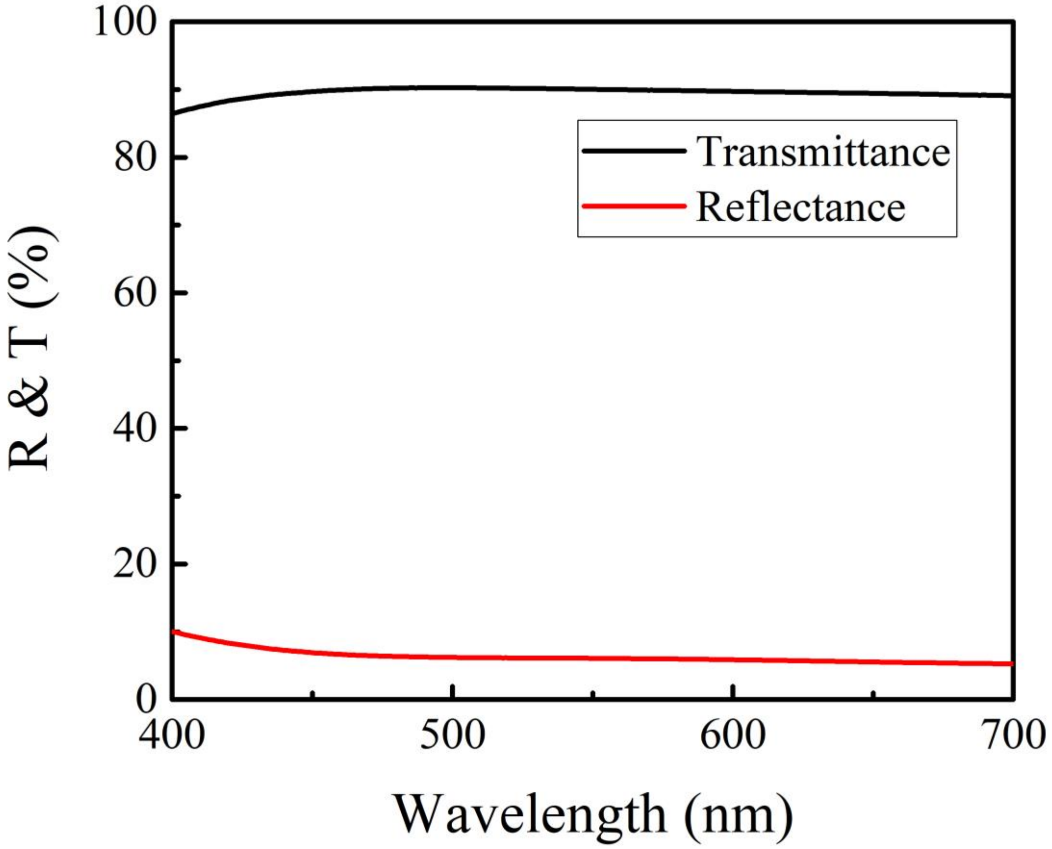

Supplementary Figure 9. Calculated absolute transmittance and reflectance spectra of the optimized structure 'PET substrate / 22 nm TiO_2 / 6.5 nm Cu-doped Ag / 72 nm Dielectric 1' across the entire visible range. Here, the refractive indices of TiO_2 and Dielectric 1 are assumed as 2.6 and 1.6, respectively. The averaged transmittance and reflectance are ~ 89.6% and ~ 6.3%, respectively.

Supplementary Figure 10

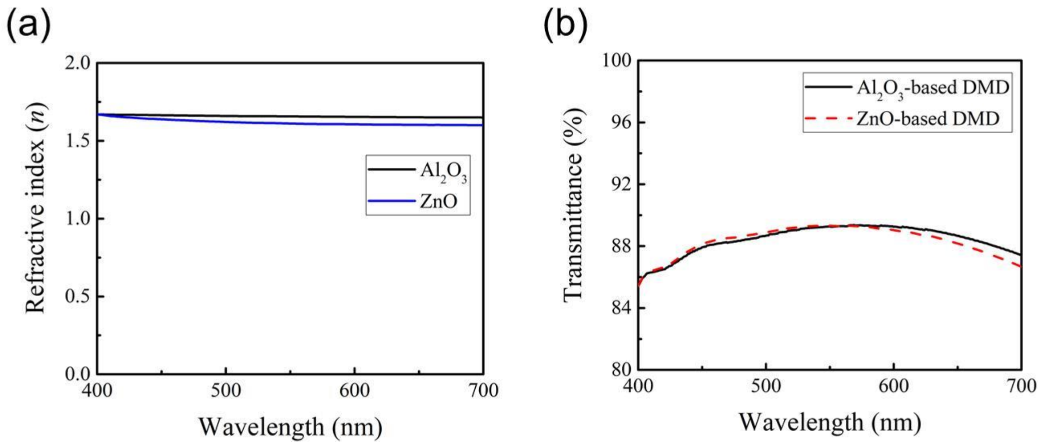

Supplementary Figure 10. DMD electrode using the ZnO sol-gel film replacing the top Al_2O_3 film. (a) The refractive index of the ZnO sol-gel film extracted from Supplementary Reference [2] compared to that of the Al_2O_3 film in this work. Both of them are very close to the ideal refractive index (n_2 = 1.65) required in our design. (b) Calculated transmittance of the DMD electrode after replacing the top Al_2O_3 with the ZnO sol-gel film. The averaged transmittance from 400 to 700 nm is ~ 88.3%, which is very close to that of the Al_2O_3-based electrode (~ 88.4%) and is higher than that of the PET substrate (~ 88.0%).

Supplementary Figure 11

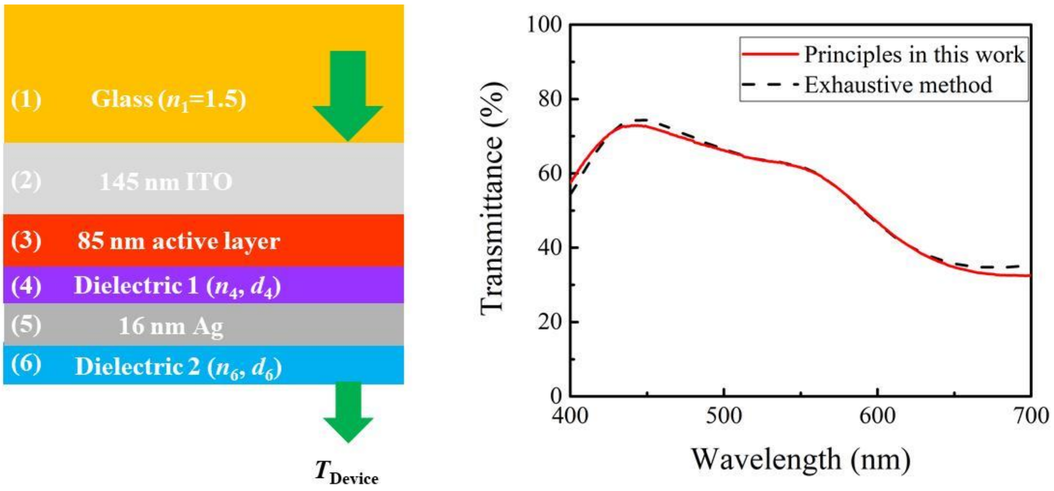

Supplementary Figure 11. Demonstration of our proposed design principles applicable for designing a DMD electrode used in a semi-transparent solar cell. The simplified, yet representative solar cell structure is shown. A ternary blend heterojunction was used as the active layer. The thicknesses of ITO, the active layer, and Ag are 145 nm, 85 nm and 16 nm, respectively. The complex refractive indices of ITO and the active layer were taken from Supplementary Reference [2] and the complex refractive index of Ag was taken from Supplementary Reference [3]. See Supplementary Reference [2] for more details on the solar cell structure. Since the active layer selectively absorbs sunlight in the red and near-infrared spectra, the transmittance of the semi-transparent solar cell averaged over the spectrum from 400 nm to 700 nm, T_{Device}, can still be used as the figure of merit for the DMD design. The transmittance spectra show that the highest averaged transmittance of the solar cell obtained by following our design principles agrees well with that obtained by exhaustively sweeping the refractive indices and thicknesses of Dielectrics 1 and 2. The highest averaged transmittance was ~ 55.4% obtained from exhaustive sweeping

the refractive indices and thicknesses of Dielectrics 1 and 2. Following our design principles, TiO_2 with recorded-high n_6=2.6 reported in Supplementary Reference [4] was used for Dielectric 2. d_6 = 24 nm obtained from Equation (9). Then n_{D1} and d_{D1} was swept to find out the optimal parameters to achieve the highest averaged transmittance. The optimal condition, where n_4 = 2.1 and d_4 = 51 nm, gives the highest averaged transmittance of ~ 54.9%, which agrees well with that designed by the exhaustive method. It is shown that the two designed transmittance spectra are nearly identical for respective methods.

Supplementary Figure 12

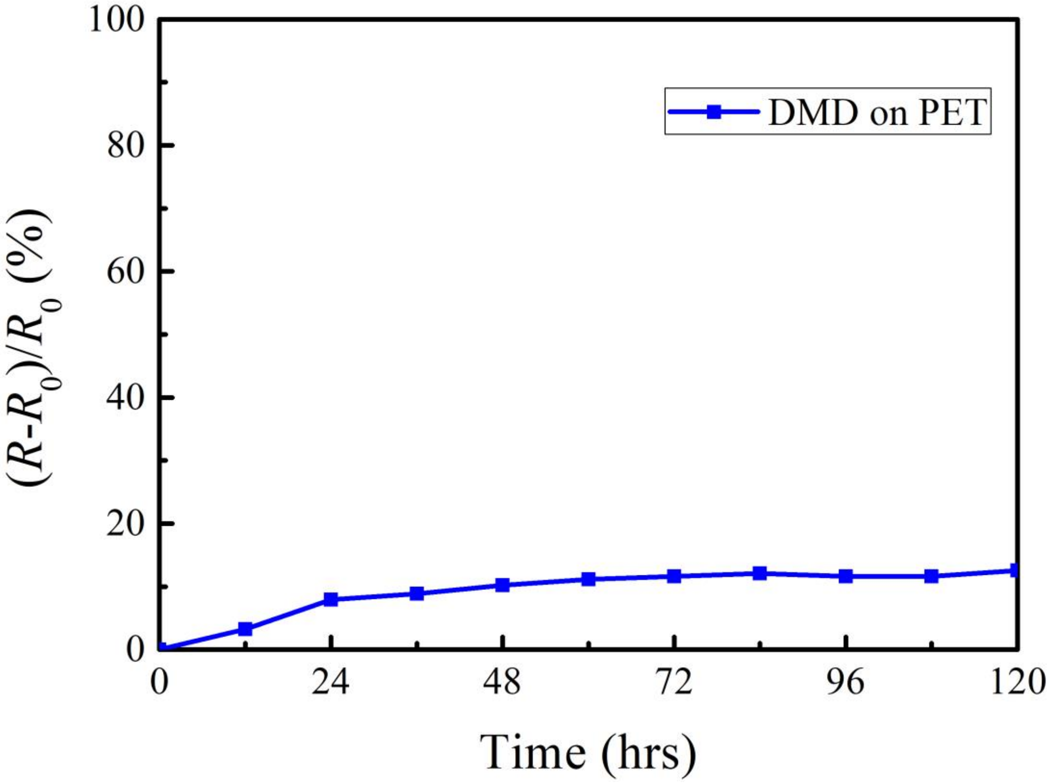

Supplementary Figure 12. Accelerated humidity test results of DMD-based transparent electrodes as a function of test duration. The test condition is 85°C and 85% relative humidity.

Supplementary References

1. Zhang, C. et al. An Ultrathin, Smooth, and Low-Loss Al-Doped Ag Film and Its Application as a Transparent Electrode in Organic Photovoltaics. *Adv. Mater.* **26**, 5696-5701 (2014).

2. Li, Y. et al. Enhanced Light Utilization in Semitransparent Organic Photovoltaics Using an Optical Outcoupling Architecture. *Adv. Mater.* **31**, 1903173 (2019).

3. Palik, E. D. *Handbook of Optical Constants of Solids* (Academic Press, Orlando, FL, 1998).

4. Maniyara, R. A., Mkhitaryan, V. K., Chen, T. L., Ghosh, D. S. & Pruneri, V. An antireflection transparent conductor with ultralow optical loss (<2 %) and electrical resistance (<6 Ω sq−1). *Nat. Commun.* **7**, 13771 (2016).
